# Evaluation of Minichromosome Maintenance-3 (MCM3) in Oral Squamous Cell Carcinoma

**Published:** 2015-06

**Authors:** Gita Rezvani, Azadeh Andisheh-Tadbir, Mohammad Javad Ashraf, Sara Amanpour, Fereshteh Kamali, Sorena Fardisi

**Affiliations:** 1 Dept. of Oral & Maxillofacial Pathology, Dental School of Shahed University, Tehran, Iran;; 2 Dept. of Oral & Maxillofacial Pathology, School of Dentistry, Shiraz University of Medical Sciences, Shiraz, Iran;; 3 Dept. of Pathology, School of Medicine, Shiraz University of Medical Sciences, Shiraz, Iran;; 4 Dept. of Oral & Maxillofacial Pathology, School of Dentistry, Kerman University of Medical Sciences, Kerman, Iran;; 5 Post Graduate Student, Dept. of Oral & Maxillofacial Pathology, School of Dentistry, Shiraz University of Medical Sciences, Shiraz, Iran;; 6 Post Graduate Student, Dept. of Oral & Maxillofacial Surgery, School of Dentistry, Shiraz University of Medical Sciences, Shiraz, Iran;

**Keywords:** MCM3, Oral Epithelial Dysplasia, Oral Squamous Cell, Carcinoma

## Abstract

**Statement of the Problem:**

The expression of minichromosome maintenance-3 (MCM3) proteins and their diagnostic value in oral mucosal dysplasia and squamous cell carcinoma (SCC) is not well known.

**Purpose:**

This study was conducted to evaluate the usefulness of minichromosome maintenance 3 (MCM3) as a biomarker for diagnosis of oral premalignant lesions and SCC.

**Materials and Method:**

In this study, 31 oral SCC, 20 dysplastic epithelium and 20 controls were selected and immunohistochemical staining was done for MCM3. ANOVA, Tukey HSD, Mann-Whitney and Kruskal-Wallis tests were used to compare the groups and the correlation between different grades.

**Results:**

There was increasing trend of MCM3 from control to dysplastic epithelium and from dysplastic epithelium to SCC both in suprabasal layers and in total epithelial layers. MCM3 expression was elevated with increasing the grade of dysplasia, but there was no statistically significant difference (*p*= 0.93). The expression was also increased in high grades of SCC compared to lower grades.

**Conclusion:**

MCM3 can be used as a useful biomarker in the diagnosis of premalignant lesions and oral SCC.

## Introduction

Proliferation markers have been broadly used to detect various human malignancies. Conventional proliferation markers such as Ki-67 and proliferation cell nuclear antigen (PCNA) are the most largely used proliferation markers in diagnosis of several human malignancies such as breast tumors, sarcoma in soft tissues, meningioma, malignancies of prostate, and non-Hodgkin lymphoma.[1-4]

Minichromosome maintenance (MCM) proteins are essential factors for replication of DNA which were initially identified in *Saccharomyces cerevisiae*.[5]MCM_2-7_ proteins are best known among this family and are critical components of the replication initiation complex which initiates synthesis of DNA in eukaryotes.[6-7]

Origin recognition complex (ORC) is a protein complex with the ability of binding to the origins of replication and forming a landing pad for the replication factors Cdc_6­_ and Cdt­_1_. At this time, MCM_s_ (MCM_2-7_) are recruited to the chromatin. So, the pre-replication complex (pre-RC) is formed which allows S-phase to be initiated. After S-phase entry, this complex is disassembled. MCM proteins and Cdc_6_ leave the chromatin following the increased activity of cyclin A-CDK_2 _(cyclin-dependent kinase2). Cdc_6_ is carried to the cytoplasm and Cdt_1_ is proteolysed. Any Cdt_1_ that has escaped proteolysis will bind to geminin.[8]

Previous experiments have demonstrated that MCM proteins 2, 4, 5, 6 and 7 are associated with several cancers.[9-12] Only a few experiments have been done on MCM_3_ compared to other members of this family. The purpose of this study was to evaluate the expression of MCM_3_ proteins and their diagnostic value in oral mucosal dysplasia and squamous cell carcinoma (SCC).

## Materials and Method

Tissue samples (n=70) were selected from the archive of oral pathology department of the Shiraz Dental School and Khalili Hospital. Twenty cases of dysplastic epithelium from lesions diagnosed as leukoplakia (including mild (n=8), moderate (n=8) and severe (n=4)) and SCC (n=31) were selected. Normal squamous epithelium was obtained from lesions diagnosed as irritation fibrosis (n=20). SCC patients had not received any chemotherapy or radiotherapy.

Hematoxylin and eosin stained slides were examined by three pathologists and cases with adequate tissues samples without hemorrhage and necrosis and similar degrees of inflammation were selected for immunohistochemistry (IHC) staining.

*Immunohistochemical Analysis*

All specimens were fixed in 10% formalin and routine histologic paraffin sections were made and stained with hematoxylin and eosin. For immunohistological analysis, the sections were cut to 3-4 mm thickness and mounted on poly-l-lysine coated slides. The sections were deparaffinized in xylene and rehydrated in alcohol. Antigen retrieval was done by Dako cytomation target retrieval solution (pH=9) in 20 minutes. Endogenous peroxidase was blocked with 3% hydrogen peroxidase/methanol. The sections were incubated with a mouse monoclonal antibody against MCM_3 _(M7263, 1:100; Dako Corporation, Denmark) as primary antibody. After that, the slides were rinsed gently with phosphate-buffered saline and an EnVision™ + Dual Link System-HRP (ready-to-use; Dako) was used as the secondary antibody. Incubation with 3, 3-diaminobenzidine tetrahydrochloride was performed for 10 min as a substrate chromogen solution (DA3 liquid k3467; Dako Corporation, Denmark). Finally, the sections were counterstained with Harris's Hematoxylin. Cervical high-grade squamous intraepithelial lesions were used as the positive control disease. Negative control was established by replacing the primary antibody with PBS.

*Immunohistochemical evaluation*

Immunohistochemical results were evaluated under a light microscope (BX41; Olympus,USA) and scored as follows; 0: no detectable staining (<5%), 1: weak but definitely detectable staining (5< and <25%), 2: moderate staining (25%< and <75%) and 3: abundant staining (>75%).[13] Staining reactions were analyzed by counting 500 cancer cells in basal layer and 500 cells in parabasal cells in each sample (original magnification X400) in addition to assessing the percentage of labeled cells in basal layer, parabasal layers and total epithelium to obtain labeling index (LI). For SCC specimens, counting 500 cells was done randomly in 5 areas of malignant epithelial nests.

*Statistical analysis*

Statistical analysis was carried out using SPSS Software, version 18. Interclass correlation coefficient (ICC) was used to evaluate the intra-observer error (ICC=0.892). ANOVA and Tukey HSD analysis were used to compare the groups. Mann-Whitney and Kruskal-Wallis test were used to evaluate the correlation between different grades. Differences were considered to be statistically significant at *p*< 0.05.

## Results

**Figure 1:**
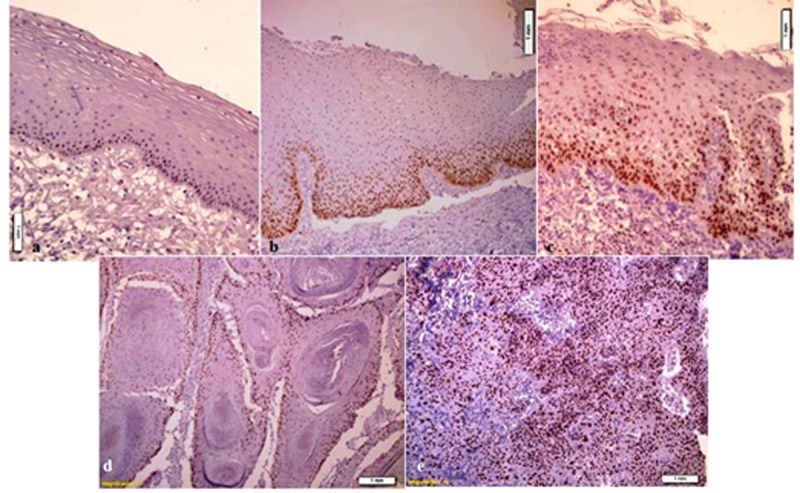
a: MCM3 immunoreactivity in normal mucosa; MCM_3_ expression is restricted to nuclei of basal cell layer and a few cells in the immediate suprabasal layers.  b: MCM3 immunoreactivity in mild epithelial dysplasia; MCM_3_ expression is restricted to the lower third of the epithelium.  c: MCM3 immunoreactivity in severe epithelial dysplasia; MCM_3 _positively stained nuclei are present throughout the epithelium.  d: MCM3 immunoreactivity in well-differentiated SCC; there are unstained foci of differentiating cells adjacent to keratin pearls.  e: MCM3 immunoreactivity in poorly differentiated SCC; MCM_3_ expression is scattered throughout the malignant epithelial cells

Clinical features of the patients are summarized in Table 1. Oral SCC specimens were composed of 31 patients with mean age of 62.6. The epithelial dysplasia consisted of 20 patients, one of which was omitted because of inadequate IHC staining, so there were 19 patients in this group with mean age of 63.5. There were also 20 patients diagnosed with irritation fibrosis in the group of normal epithelium. One sample was omitted due to folding of tissue section and difficulty of cell counting; therefore, this category consisted of 19 cases with mean age of 46.2. All samples were positive for MCM_3_ staining. In cases of normal epithelium, MCM_3_ expression was restricted to the nuclei of basal cell layer and a few cells in the immediate suprabasal layers. The superficial differentiating cells were negative in normal epithelium. In dysplastic epithelium, MCM_3 _positively stained nuclei were present above the basal layer, whereas in SCC samples, nuclear staining was seen in superficial layers (Figure 1).

**Table 1:** Age and gender distribution of the patients

	**Age**	**Sex**		**Grade**
		**Female**	**Male**	
SCC	12 (38.7%)	19 (61.3%)	.313±62.3	20= I (Well. diff.)
				9= II (Mod. diff.)
				2= III (Poorly. diff.)
Dysplasia	12(63.2%)	7(36.8%)	15.6±63.5	8= Mild
				8= Mod.
				3= Severe
Normal	15(78.9%)	4(21.1%)	16.9±46.2	

Labeling Index in basal cell layer of normal epithelium and dysplastic samples was 57.45 (±21.84) and 68.87 (±25.34), respectively, with no statistically significant difference (*p*= 0.153). Likewise, no significant difference was observed either between the LI in basal cells of normal epithelium and SCC (*p*= 0.152) or the epithelial dysplasia and SCC (*p*= 0.153). MCM_3_ labeling index in suprabasal cell layers of normal and dysplastic epithelium (18.20±9.57 and 38.83±18.89 respectively) had significant difference (*p*= 0.001). There was also a significant difference between the suprabasal layer of dysplastic epithelium and SCC (*p*= 0.007).

**Figure 2:**
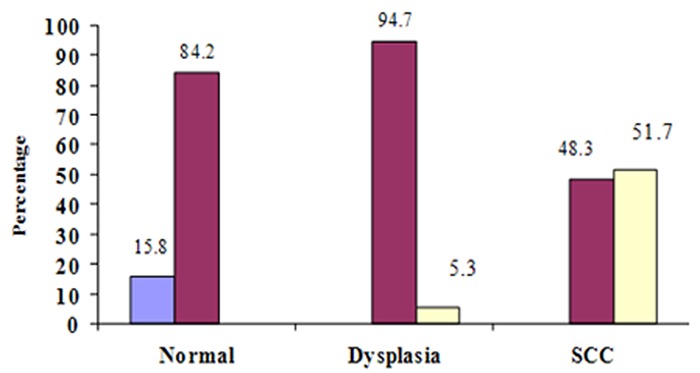
Score staining in different groups

The LI of total epithelial layers of normal epithelium, dysplastic epithelium and SCC was 37.88±12.93, 51.64±17.70 and 67.93±15.91, respectively. Comparative analysis showed significant difference between them (*p*= 0.001). In cases of well-differentiated SCC, there were unstained foci of differentiating cells adjacent to keratin pearls, whereas in moderately and poorly differentiated SCC, MCM_3_ expression was scattered throughout the malignant epithelial nests. In normal epithelium samples, 16 cases had moderate staining and 3 cases showed weak staining. Out of the dysplastic epithelium samples, 18 cases showed moderate and 1 case severe staining. In SCC group, 16 cases had severe and 15 cases showed moderate staining (Figure 2). Score staining showed no significant difference between dysplastic epithelium and SCC (*p*= 0.11) and also between normal epithelium and SCC (*p*= 0.10).

**Figure 3:**
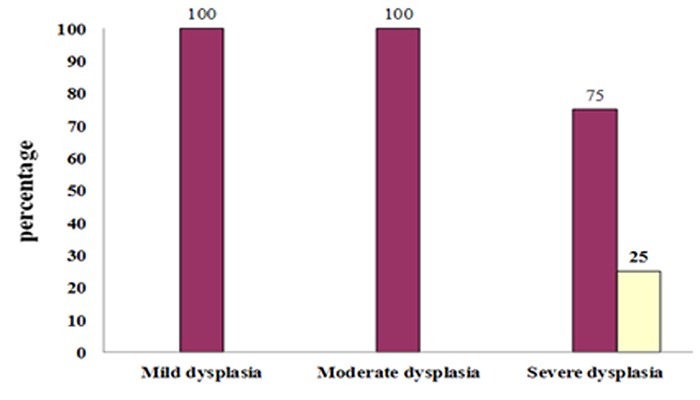
Score staining in dysplastic lesions

In samples of dysplastic epithelium, MCM_3 _expression increased gradually and became stronger from mild dysplasia to severe dysplasia (Figure 3) but did not have significant difference (*p*= 0.93).

**Figure 4:**
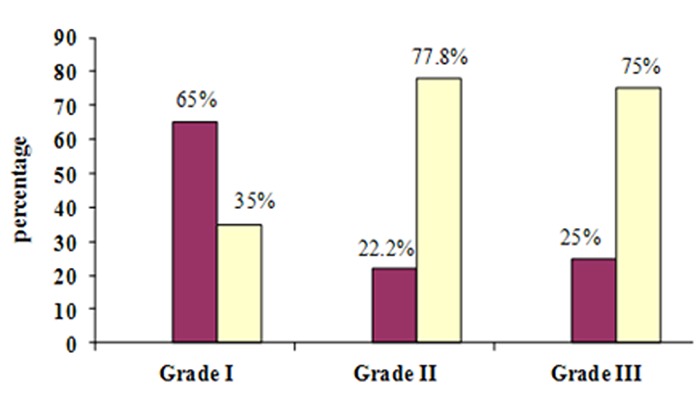
Score staining in different grades of SCC

In SCC cases, the expression of MCM_3_ protein gradually increased (Figure 4) and became significantly stronger from poorly differentiated lesions to well-differentiated SCC (*p*= 0.04). The only significant correlation between grades of dysplasia and score staining was found in grade III of dysplasia (*p*= 0.001). No Significant correlation was found between different grades of SCC and score staining (*p*= 0.46).

## Discussion

Squamous cell carcinoma of oral cavity is a common malignant lesion of head and neck, especially in developing countries because of high exposure of large population to prevalent carcinogens such as tobacco smoke and betel nut.[14] The nonspecific clinical appearance of dysplastic and malignant lesions of oral cavity in early stages, emphasize the importance of finding effective and suitable methods for detection of such lesions in early stages. Ectopic cell cycle entry is a main feature of dysplasia and malignancy. MCM_3_ is a member of minichromosome maintenance protein family with a critical role in initiation of DNA replication.[14] It is present during cellular proliferation of normal cells, premalignant and neoplastic cells but absent in cells that are in G0 phase.[9] The fundamental role of MCM proteins in DNA replication and their diminishing role in quiescent cells may give the MCM proteins the function of a proliferative marker in cancer screening.[13] Ha *et al.* examined MCM_3_ expression in a variety of human tumors including lymphoma, leukemia, carcinomas of the cervix, breast, kidney, stomach, lung, colon and malignant melanoma.[15]Immunohistochemical analysis and western blot revealed that expression of MCM_3_ protein had increased in most of the human cancer tissues tested. They compared the expression of MCM_3_ proteins in human malignancies with conventional proliferation markers, proliferating cell nuclear antigen (PCNA) and Ki67. MCM_3_ antibody was most specific for multiple human cancers, whereas PCNA had less specificity and Ki67 could not detect several human cancers.[15] In another study, Endle *et al.* evaluated the expression pattern of MCM_3_, P_27 _and Ki67 proteins on germinal centers and oral mucosa. The expression of p27 protein was related to differentiated cells, while localization of MCM3 and Ki67 was mainly in regions of proliferating cells.[16]Gan *et al.* evaluated the expression of MCM_3_ and MCM_4_ proteins in cervical SCC and found that MCM3 and MCM4 expression had a tendency to be stronger from control group to cervical SCC. Both MCM_3_ and MCM_4_ were significantly up regulated in cervical SCC compared with the control group, CIN grade 1 and grade 2-3. MCM_3_ expression was correlated with cervical SCC cell differentiation.[13] Nonetheless, the expression level and usefulness of MCM_3_ as a proliferative marker in patients with SCC of oral cavity have to be explained well, yet. In this study the expression of MCM_3_ in oral dysplastic epithelium and SCC was examined. Basal cells in all specimens were positive for MCM_3_. Recently, Takeda *et al.* evaluated the expression of stem cell markers such as cytokeratin 19 and p 63 and proposed that stem cells are located in basal layer of normal mucosa in oral cavity.[17]In normal oral mucosa, most cells are not in cell cycle but in G0 phase. A smaller number of cells are also in G0-G1 transition. The amount of cells which are actually in the cell cycle is only 20%.[18]

As MCM3 proteins can be detected both in cells which are in cell cycle and cells in G0-G1 transition, MCM_3_ expression level in basal cells of normal, dysplastic and SCC specimens had no significant difference. MCM3 expression showed a tendency to be stronger from normal epithelium to SCC. In normal epithelium, the staining was limited to the nuclei of basal cells and a few cells in the immediate suprabasal layers. In dysplastic epithelium, MCM3 was expressed in the lower and middle thirds of epithelium. In SCC, there was wide spread expression of MCM3 in full thickness of malignant epithelium. The expression of MCM3 in epithelial nests was limited to the periphery in well differentiated SCC but was scattered in moderate and poorly differentiated SCC. These finding were in agreement with the results of Chatrath *et al.*,[19] Gan *et al.*[13] and Freeman *et al.*[20] The more differentiated the cells, the less proliferation activity is seen, so the expression of MCM3 proteins is reduced. For this reason, in normal epithelium, the expression is limited to the basal cell layer and very few cells in parabasal layers; however, in dysplastic samples more cells are in the cell cycle. Due to loss of cell cycle control mechanisms in malignant cases, a greater number of cells and in more upper layers of the epithelium were positive for MCM3. In well-differentiated SCC, due to terminal differentiation of cells in areas of keratin pearl formation, these cells were negative for staining. These results were in agreement with the results of Torres-Rendon *et al.*[18] and Freeman *et al.*[20] Positive expression in total epithelial layers was also in increasing order from normal epithelium to SCC with maximum expression in SCC. In dysplastic epithelium, there was a direct correlation between grades of dysplasia and MCM3 expression. With increase in grades of the lesion, MCM3 expression and staining score have increased as well. Torres-Rendon *et al.*[18] and Kodani *et al.*[10] also reported a positive correlation between dysplastic grades and MCM3 expression. Ibarra *et al.* showed in 2008 that a complete complex of MCM is necessary to protect the integrity of genome against natural replication stress during S phase.[21] So, the higher expression of MCM3 in higher grades of dysplastic in our study may propose the same defensive mechanism against genomic injury and before malignant transformation.[18] In SCC, most of the differentiated tumor cells showed higher expression and score staining for MCM3 similar to the result of Gan *et al.*’s study in 2010,[13] which showed positive correlation between the tumor differentiation and MCM3 expression in cervical SCC. They also reported higher expression of MCM3 in poorly differentiated lesions.

## Conclusion

In conclusion, findings of the present study showed that MCM3 could be used as a useful marker in diagnosis of premalignant lesions and SCC of oral cavity. 

## References

[1] Jansen RL, Hupperets PS, Arends JW, Joosten Achjanie SR, Volovics A, Schouten HC (1998). MIB-1 labelling index is an independent prognostic marker in primary breast cancer. Br J Cancer.

[2] Perry A, Stafford SL, Scheithauer BW, Suman VJ, Lohse CM (1998). The prognostic significance of MIB-1, p53, and DNA flow cytometry in completely resected primary meningiomas. Cancer.

[3] Mashal RD, Lester S, Corless C, Richie JP, Chandra R, Propert KJ, Dutta A (1996). Expression of cell cycle-regulated proteins in prostate cancer. Cancer Res.

[4] Gerdes J, Dallenbach F, Lennert K, Lemke H, Stein H (1984). Growth fractions in malignant non-Hodgkin's lymphomas (NHL) as determined in situ with the monoclonal antibody Ki-67. Hematol Oncol.

[5] Maine GT, Sinha P, Tye BK (1984). Mutants of S. cerevisiae defective in the maintenance of minichromosomes. Genetics.

[6] Tye BK (1999). Minichromosome maintenance as a genetic assay for defects in DNA replication. Methods.

[7] Chong JP, Thömmes P, Blow JJ (1996). The role of MCM/P1 proteins in the licensing of DNA replication. Trends Biochem Sci.

[8] Tye BK (1999). MCM proteins in DNA replication. Annu Rev Biochem.

[9] Gonzalez MA, Tachibana KE, Laskey RA, Coleman N (2005). Control of DNA replication and its potential clinical exploitation. Nat Rev Cancer.

[10] Kodani I, Shomori K, Osaki M, Kuratate I, Ryoke K, Ito H (2001). Expression of minichromosome maintenance 2 (MCM2), Ki-67, and cell-cycle-related molecules, and apoptosis in the normal-dysplasia-carcinoma sequence of the oral mucosa. Pathobiology.

[11] Tan DF, Huberman JA, Hyland A, Loewen GM, Brooks JS, Beck AF (2001). MCM2--a promising marker for premalignant lesions of the lung: a cohort study. BMC Cancer.

[12] Going JJ, Keith WN, Neilson L, Stoeber K, Stuart RC, Williams GH (2002). Aberrant expression of minichromosome maintenance proteins 2 and 5, and Ki-67 in dysplastic squamous oesophageal epithelium and Barrett's mucosa. Gut.

[13] Gan N, Du Y, Zhang W, Zhou J (2010). Increase of Mcm3 and Mcm4 expression in cervical squamous cell carcinomas. Eur J Gynaecol Oncol.

[14] Scott IS, Odell E, Chatrath P, Morris LS, Davies RJ, Vowler SL (2006). A minimally invasive immunocytochemical approach to early detection of oral squamous cell carcinoma and dysplasia. Br J Cancer.

[15] Ha SA, Shin SM, Namkoong H, Lee H, Cho GW, Hur SY (2004). Cancer-associated expression of minichromos-ome maintenance 3 gene in several human cancers and its involvement in tumorigenesis. Clin Cancer Res.

[16] Endl E, Kausch I, Baack M, Knippers R, Gerdes J, Scholzen T (2001). The expression of Ki-67, MCM3, and p27 defines distinct subsets of proliferating, resting, and differentiated cells. J Pathol.

[17] Takeda T, Sugihara K, Hirayama Y, Hirano M, Tanuma JI, Semba I (2006). Immunohistological evaluation of Ki-67, p63, CK19 and p53 expression in oral epithelial dysplasias. J Oral Pathol Med.

[18] Torres Rendon A, Roy S, Craig GT, Speight PM (2009). Expression of Mcm2, geminin and Ki67 in normal oral mucosa, oral epithelial dysplasias and their corresponding squamous-cell carcinomas. Br J Cancer.

[19] Chatrath P, Scott IS, Morris LS, Davies RJ, Rushbrook SM, Bird K (2003). Aberrant expression of minichromoso-me maintenance protein-2 and Ki67 in laryngeal squamous epithelial lesions. Br J Cancer.

[20] Freeman A, Morris LS, Mills AD, Stoeber K, Laskey RA, Williams GH (1999). Minichromosome maintenance proteins as biological markers of dysplasia and malignancy. Clin Cancer Res.

[21] Ibarra A, Schwob E, Méndez J (2008). Excess MCM proteins protect human cells from replicative stress by licensing backup origins of replication. Proc Natl Acad Sci U S A.

